# Effects of sodium on nitrogen evolution characteristics during the fast co-pyrolysis of coal and biomass

**DOI:** 10.1039/d6ra07143k

**Published:** 2026-09-28

**Authors:** Zhihao Huang, Shicai Kuang, Bo Niu, Yue Dang, Fuxiang Liu, Qi Yu, Shiyu Gong, Sheng Su

**Affiliations:** a Datang Northwest Electric Power Test and Research Institute Co., Ltd Xi'an Shaanxi 710018 China huangzhihao_13@163.com; b State Key Laboratory of Coal Combustion, School of Energy and Power Engineering, Huazhong University of Science and Technology Wuhan 430074 China

## Abstract

The co-combustion of biomass and coal is a crucial initiative for advancing carbon neutrality. However, the specific influence of alkali metals on nitrogen evolution during fast co-pyrolysis remains unclear. This study investigates the regulatory effects of sodium (Na) on the migration and transformation of volatile-N and char-N during the rapid pyrolysis and co-pyrolysis of sawdust (SD) and lignite coal (LC). Acid-washed and Na-loaded samples, including individual SD/LC and blended samples (blending ratios of 5% and 15%), were pyrolyzed in a vertical fixed-bed reactor at 500 °C and 800 °C. The evolution of volatile-N (measured as NH_3_ and HCN) and char-N functional groups was analyzed using an FTIR gas analyzer and employing X-ray photoelectron and Raman spectroscopies. Results show that at low temperatures of 500 °C, labile biomass N-A decomposes into NH_3_, while coal N-5 and N-6 remain relatively stable. At 800 °C, heterocyclic nitrogen undergoes ring-opening to form HCN, while coal preferentially retains nitrogen as N-Q. Crucially, Na addition alters these pathways, promoting the apparent NH_3_ and HCN release from biomass at 500 °C while inhibiting heterocyclic ring-opening and N-Q hydrogenation at 800 °C. During co-pyrolysis, Na consistently suppresses volatile-N precursors by inhibiting the decomposition of heterocyclic nitrogen and promoting char-N stabilization mainly as N-5 and N-Q. Raman analysis further reveals that Na regulated the evolution of aromatic carbon structures in blended chars, thereby affecting char-N retention within the carbon matrix. These findings provide valuable insights into controlling NO_*x*_ precursor emissions during the fast co-pyrolysis of coal and biomass through alkali metal management.

## Abstract 2

Sodium suppresses NH_3_ and HCN release during the co-pyrolysis of coal and biomass by hindering the polycondensation of small aromatic rings and redistributing char nitrogen towards pyrrolic and quaternary forms.

## Graphical abstract

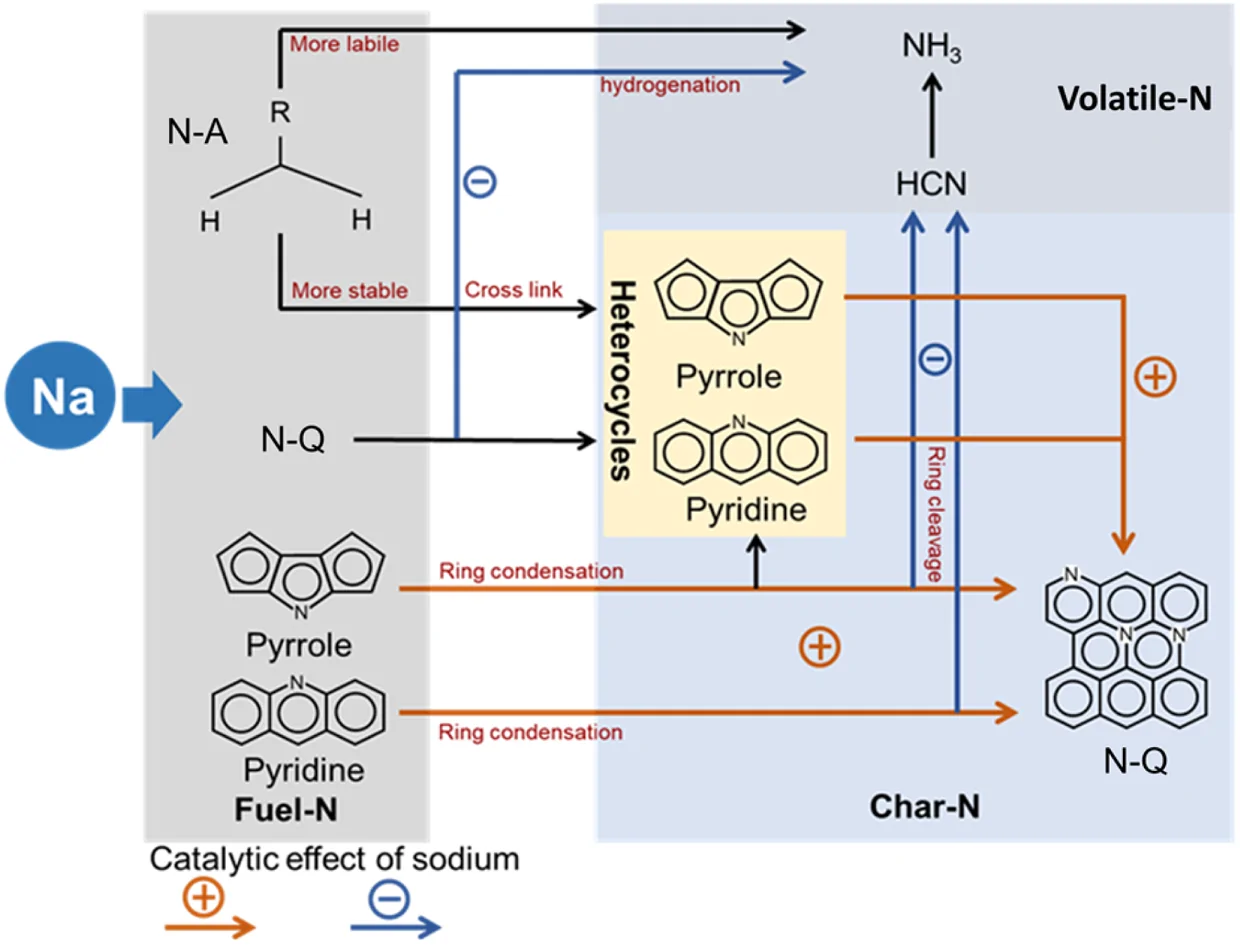

## 1. Introduction

Amidst the global transition toward sustainable energy systems, biomass has emerged as an indispensable carbon-neutral resource, offering a viable pathway to mitigate the global greenhouse effect and promote resource circularity.^1,2^ Its valorization can proceed either through catalytic upgrading, in which the biomass-derived platform molecules are selectively converted into liquid biofuels and fine chemicals over tailored solid catalytic materials,^3–5^ or through thermochemical conversion, in which the whole feedstock is converted directly into heat and power. The latter remains the dominant route for large-scale deployment. Despite its environmental advantages, the large-scale deployment of biomass as a primary monofuel is severely constrained by its intrinsic physicochemical limitations, most notably its low energy density, high moisture content, and seasonal supply fluctuations.^6,7^ These drawbacks necessitate the co-firing of biomass with traditional coal as a pragmatic and techno-economically viable strategy.^8^ In coal-fired power plants, this integrated approach not only enhances the energy utilization of biomass but also significantly reduces the overall NO_*x*_ emissions owing to the inherent low sulfur/nitrogen content of biomass.^9^ Notably, pyrolysis represents the foundational stage in the thermochemical conversion of solid fuels as it precedes and dictates the subsequent gas-phase and heterogeneous combustion reactions. Fundamental to this stage is the evolution of fuel-bound nitrogen (fuel-N), which governs the initial devolatilization (volatile-N) and char formation (char-N) during pyrolysis, playing a vital role in the ultimate formation and reduction of NO_*x*_ emissions.^10^ Therefore, elucidating the fuel-N migration and transformation pathways during the co-pyrolysis of coal and biomass is a critical prerequisite for developing precision-controlled, low-emission combustion technologies.

In fact, pyrolysis is a sophisticated process wherein the partitioning of fuel-N is modulated by multiple factors. While nitrogen evolution during the pyrolysis of individual biomass components has been extensively studied, scientific consensus remains elusive.^11^ Currently, NH_3_ and HCN are universally recognized as the primary gaseous NO_*x*_ precursors, with HCN typically prevailing under high-heating-rate conditions due to its favorable formation kinetics from the cleavage of heterocyclic nitrogen functionalities.^12,13^ Given that proteins are the predominant nitrogen-containing components in biomass, researchers have utilized amino acids or their pyrolytic derivatives as model compounds to decipher these pathways. These studies have highlighted the critical catalytic influence of alkali and alkaline earth metals (AAEMs) on nitrogen migration.^14^ For example, Wei *et al.*^15^ utilized TG-FTIR to demonstrate that K, Na, Ca, and Mg inhibited the formation of NH_3_, HCN, and HNCO during glutamic acid pyrolysis. In contrast, Zhou *et al.*^16^ reported that K^+^ and Na^+^ promote NH_3_ formation while inhibiting HCN, the primary gaseous nitrogen product. In addition, density functional theory (DFT) studies by Zhang *et al.*^17^ and Liu *et al.*^18^ further revealed that K^+^ and Na^+^ primarily lower the activation energy barriers for nitrogen-containing ring decomposition, thereby accelerating volatile-N release. Overall, existing research is insufficient to elucidate complex nitrogen transformation and migration in actual pyrolysis environments, which remains a subject of intense debate. In the scenario of biomass and coal co-pyrolysis, the underlying mechanisms are even more ambiguous. Lin *et al.*^19^ and Yuan *et al.*^20^ observed that the co-pyrolysis of sub-bituminous coal and wheat straw led to a reduction in NH_3_ release, but an increase in HCN emission. Considering the significant impact of alkali metals on single-feedstock pyrolysis, the variations in gaseous and solid nitrogen precursors during co-pyrolysis are likely driven by the abundant alkali metals (predominantly soluble K and Na) present in the biomass.^15,21^ Nevertheless, a systematic elucidation of how specific alkali metals (*e.g.*, Na) mechanistically influence the transformation and migration of gas/solid-phase nitrogen during biomass and coal co-pyrolysis is still lacking, presenting a critical gap between fundamental molecular theories and complex industrial applications.

Herein, this work elucidates the mechanistic influence of alkali metals (Na) on nitrogen transformation and migration during fast co-pyrolysis of biomass and coal. Using sawdust (SD) and lignite coal (LC) as feedstocks in a vertical fixed-bed reactor, we systematically compared the generation of volatile-N between acid-washed (demineralized) and alkali (Na)-loaded samples at a range of temperatures. Furthermore, X-ray photoelectron spectroscopy (XPS) was employed to characterize the effects of the alkali metal Na on the speciation and abundance of char-N functionalities, while Raman spectroscopy was applied to probe the evolution of char carbon skeletons, especially the relative proportions of small and large aromatic ring structures. By correlating volatile-N release, char-N speciation, and carbon structural parameters from Raman spectroscopy, this study aims to clarify how Na regulates nitrogen retention and transformation during the co-pyrolysis of coal and biomass. The results demonstrate that Na promotes the release of NH_3_ and HCN during the pyrolysis of SD at 500 °C but inhibits it at 800 °C, whereas for LC and all blended samples, Na suppresses the release of both species at 500 °C and 800 °C. This study is expected to provide critical theoretical insights for optimizing biomass-coal co-combustion technologies and achieving the reduction of NO_*x*_ emissions at the source.

## 2. Experimental methods

### 2.1 Sample preparation and characterization

The biomass feedstock selected for this study was sawdust (SD, original), and the coal sample was lignite coal (LC, original). Both raw materials were ground and sieved to collect particles in the size range of 150–180 µm. The samples were subsequently dried in an oven at 105 °C for 12 h. The proximate and ultimate analyses of the raw samples are presented in Table 1 and were conducted in accordance with Chinese national standards GB/T212-2008 and GB/T31391-2015. The sodium (Na) content in the raw materials was quantified using ion chromatography (IC, DIONEX AQ-1100), yielding 0.27% for SD-original and 2.75% for LC-original. To remove alkali metals, a demineralization process was applied to both the coal and biomass. A 50 g sample was mixed with 500 mL of 3 mol L^−1^ hydrochloric acid in a beaker. The mixture was stirred using a magnetic stirrer in a water bath at 60 °C for 24 h. The residue was then filtered *via* vacuum filtration and washed repeatedly with deionized water until the filtrate reached a neutral pH (∼7). Finally, the samples were dried at 105 °C for 24 h to obtain the demineralized samples. The proximate and ultimate analysis results for these samples (SD/LC) are also shown in Table 1.

**Table 1:** Proximate and ultimate analysis results

Samples	Proximate wt/%				Ultimate wt/%				
	*M*_ad_	*A*_ad_	*V*_ad_	FC_ad_	*C*_ad_	*H*_ad_	*N*_ad_	*S*_ad_	*O*_ad_ a
SD, original	7.02	0.88	77.45	14.65	50.64	5.13	0.67	0.03	35.63
LC, original	1.53	7.02	44.92	46.53	69.96	4.42	0.96	0.95	15.15
SD	4.39	0.12	84.49	11.00	51.98	5.31	0.77	0.02	37.41
LC	4.06	1.48	44.47	49.99	68.41	4.04	1.54	0.54	19.93

a The oxygen content is calculated by the difference.

Sodium-loaded biomass was prepared using a wet impregnation method. Specifically, NaCl was mixed with the acid-washed biomass at a ratio of 0.02 g g^−1^ and dissolved in deionized water. The solution was then subjected to rotary evaporation. Upon drying, a biomass sample impregnated with 2 wt% NaCl was obtained. All samples used for pyrolysis in this study underwent acid washing. This corresponds to approximately 0.79 wt% Na, about 3 times the content originally present in the untreated sawdust and well below that of the untreated coal, so that the catalytic effect is resolvable. The acid-washed sample loaded with Na was designated as SD-Na. Blends were prepared by mechanically mixing biomass with coal at mass percentages of 5% and 15%; the acid-washed blends were labeled 5SD/LC and 15SD/LC, while the blends containing Na-loaded biomass were designated as 5SD/LC-Na and 15SD/LC-Na.

### 2.2 Pyrolysis system and conditions

The pyrolysis experiments were conducted using the vertical fixed-bed reactor system illustrated in Fig. 1. The system comprises three main units: gas supply, pyrolysis reactor, and flue gas detection. N_2_ was used as the carrier gas, with the flow rate maintained at 3 L min^−1^ by a mass flow controller. The evolved pyrolysis gas passed through a cooling unit, a drying tube packed with anhydrous CaCl_2_, and a heating element to the analyzer. Finally, the concentrations of NH_3_ and HCN in the flue gas were monitored online using a Gasmet DX4000 Fourier transform infrared (FTIR) gas analyzer. As all samples were measured using an identical system, gas-product losses during the condensation and drying processes apply to each measurement and do not affect comparisons between samples on which this work is based.

**Fig. 1:**
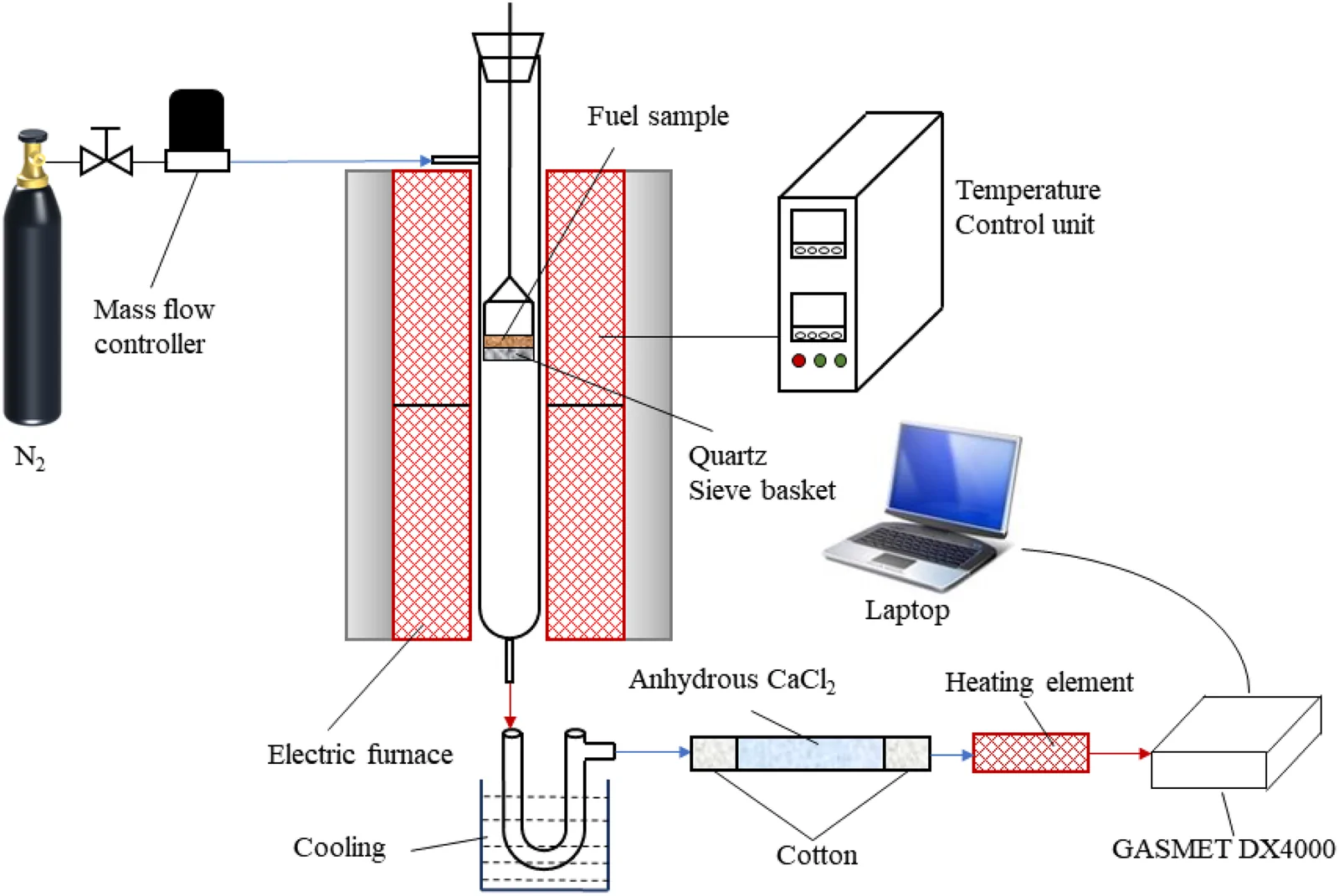
Schematic of the fixed-bed pyrolysis system.

For each experiment, 0.5 g (±0.005 g) sample was loaded into a quartz basket and initially positioned in the low-temperature zone outside the furnace. The reactor was heated to 500 °C or 800 °C at a rate of 20 °C min^−1^, during which the system was purged with N_2_ for 300 s. The basket was then rapidly inserted into the constant-temperature reaction zone and maintained there for 20 min. The chars obtained after pyrolysis at 500 °C were denoted as SD_500_, LC_500_, SD-Na_500_, 5SD/LC_500_, 5SD/LC-Na_500_, 15SD/LC_500_, and 15SD/LC-Na_500_. Correspondingly, chars obtained at 800 °C were labeled with the suffix “800” (*e.g.*, SD_800_ and LC_800_).

### 2.3 Product analysis

During pyrolysis, fuel-N is partitioned among NO_*x*_ precursors, tar-bound nitrogen, N_2_ and char-N.^22^ Since NH_3_ and HCN are the two species that are subsequently oxidized to NO_*x*_ in the combustion zone, they were selected as the typical N-containing products and quantified by online FTIR gas analysis. The term volatile-N is used hereafter to denote the sum of these two species in this work. The distribution of char-N functionalities was determined by X-ray photoelectron spectroscopy (XPS) N 1s deconvolution, which resolves the relative speciation of char-N. This combination of online FTIR and XPS has been widely adopted to examine nitrogen evolution during rapid pyrolysis of solid fuels in fixed-bed reactors^23^ and was adopted here, as the objective is to identify the nitrogen functionalities through which Na redirects conversion pathways rather than to partition fuel-N among all products. Tar-bound nitrogen and N_2_ were not determined, as their quantification is not accessible with the present system.

XPS (AXIS-ULTRA DLD, Kratos, Japan) was employed to analyze the raw samples and pyrolysis chars. The different electron binding energies of the N 1s orbital indicate distinct chemical bonds. In this study, the binding energies for nitrogen-containing components were assigned as follows: 398.7 ± 0.3 eV for pyridinic nitrogen (N-6), 399.7 ± 0.4 eV for amine/amide nitrogen (N-A), 400.3 ± 0.2 eV for pyrrolic nitrogen (N-5), 401.4 ± 0.3 eV for quaternary nitrogen (N-Q), and 403.5 ± 0.5 eV for oxidized nitrogen species (N-X).^24^

The distribution of nitrogen functional groups in the raw samples and pyrolysis chars was calculated as eqn (1):1
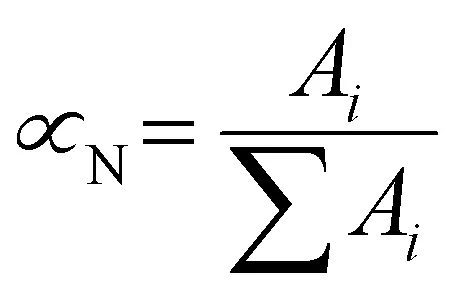
where *∝*_N_ is the ratio of a specific nitrogen functional group, and *A*_*i*_ is the peak area of the corresponding functional group in the XPS spectrum.

The yield of rapid pyrolysis gas products was calculated using eqn (2):2
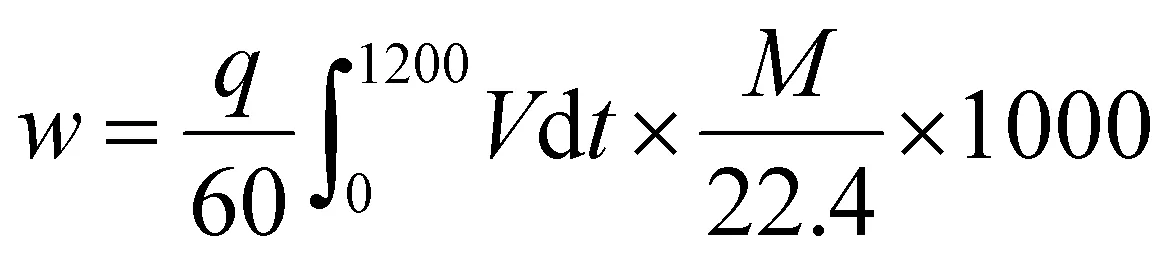
where *w* is the yield of the gas product, *q* is the gas flow rate (L min^−1^), and *V* is the real-time volume fraction of the gas (ppm); 1200 represents the total pyrolysis duration (s), *M* is the molar mass of the gas molecule (g mol^−1^), and 22.4 is the molar volume of an ideal gas (L mol^−1^).

The mass fraction of gaseous nitrogen was calculated using eqn (3):3
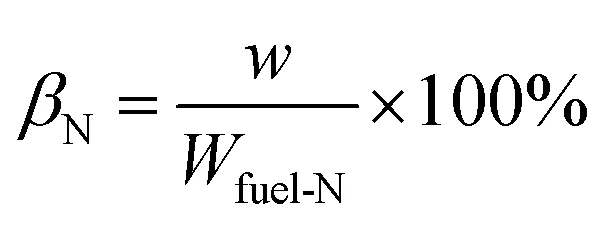
where *W*_fuel-N_ is the mass of nitrogen in the fuel (g), and *β*_N_ is the mass fraction of pyrolysis gas nitrogen relative to total nitrogen in the fuel. Specifically, *β*_N_ is an apparent conversion ratio for NH_3_ and HCN.

### 2.4 Raman spectroscopy analysis

Raman analysis of the samples was performed using a confocal Raman microscope (Thermo Fisher DXR2). The first-order Raman spectra of coal and biomass chars typically exhibit two characteristic bands: the G band and the D band. Following the ten-peak fitting method established for coal chars, a ten-peak fitting method was used to deconstruct the Raman spectra, and the relative intensities of the fitted peaks were used to characterize the relative content of various structures in the sample chars^25,26^ (Fig. 2).

**Fig. 2:**
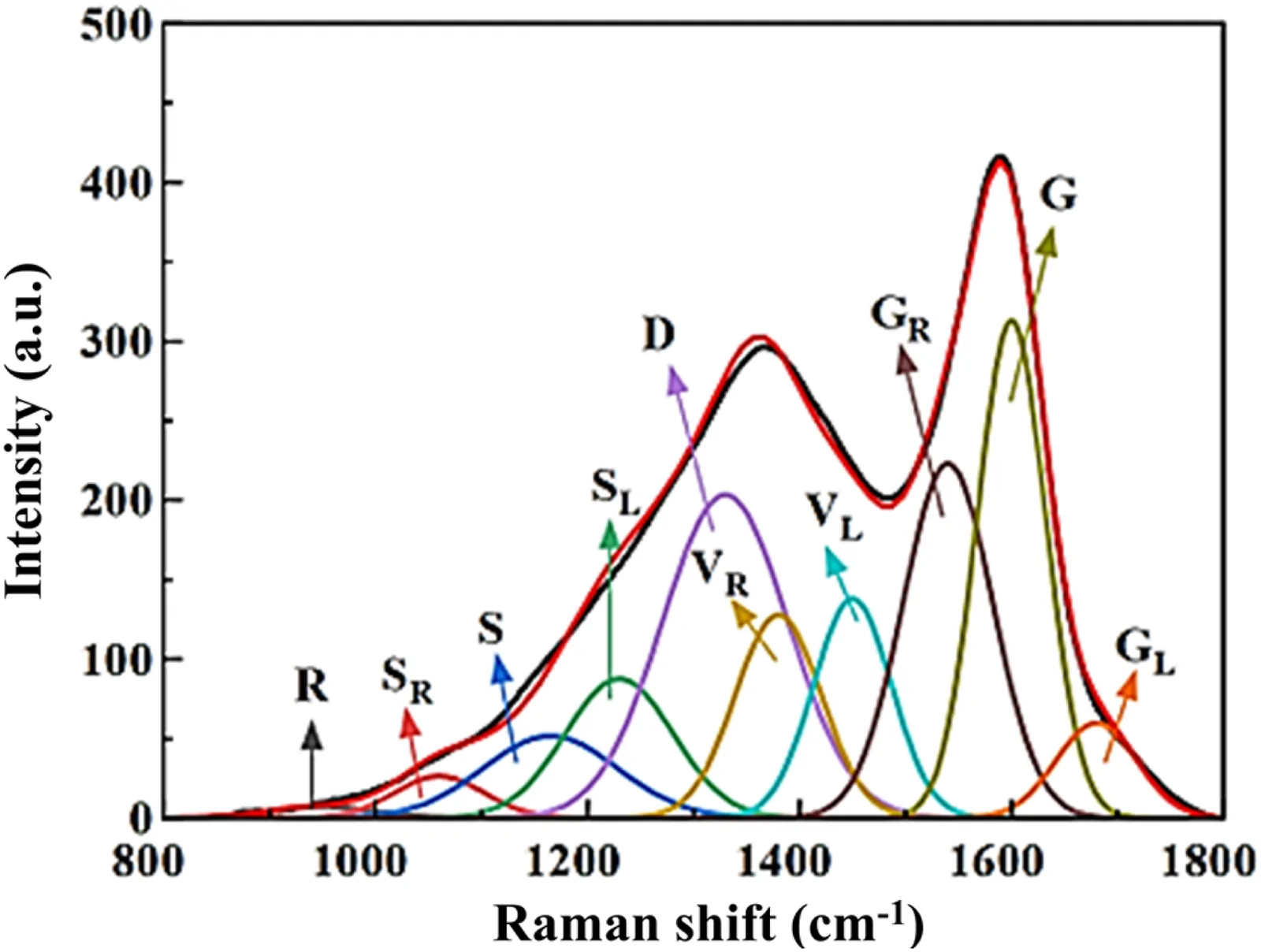
Schematic of the ten-peak fitted Raman spectra.

In this method, the V_R_, V_L_, and G_R_ peaks represent amorphous carbon structures and small aromatic ring structures. The D peak represents the C–C structures between aromatic systems containing not less than six rings.^27^ The *A*_V_R_+V_L_+G_R__, *A*_D_, and *A*_all_ represent the peak areas of amorphous carbon, large aromatic rings, and the total peak area, respectively. The parameters *A*_V_R_+V_L_+G_R__/*A*_D_ and *A*_V_R_+V_L_+G_R__/*A*_all_ reflect the ratio of small aromatic rings to large aromatic rings and the relative content of small aromatic rings, respectively.

## 3. Results and discussion

### 3.1 Nitrogen evolution characteristics during individual pyrolysis of biomass/coal

#### 3.1.1 Volatile-N transformations

During fast pyrolysis of biomass and coal, fuel-N is transformed into various volatile nitrogen (volatile-N) species, including NH_3_, HCN, N_2_, HNCO, and NO. Since extensive studies^28–30^ have identified NH_3_ and HCN as the primary volatile-N precursors of NO_*x*_ during the pyrolysis of coal and biomass, this work specifically focused on their generation.

Fig. 3 illustrates the evolution profiles of apparent NH_3_ and HCN during rapid individual pyrolysis of SD and LC. At a pyrolysis temperature of 500 °C, the conversion of fuel-N to NH_3_ was significantly higher than that to HCN for SD. In contrast, LC exhibited generally low conversion rates for both NH_3_ and HCN. At 800 °C, the conversion to HCN surpassed that of NH_3_ for both SD and LC. Additionally, the HCN yield from SD was higher than that from LC, whereas its NH_3_ yield was relatively lower. These phenomena can be attributed to differences in fuel characteristics and the redistribution of nitrogen functional groups induced by temperature variations. During low-temperature pyrolysis (500 °C), the thermally unstable N-A present in biomass preferentially decomposes to release NH_3_, while the stable pyrrolic and pyridinic nitrogen forms in coal are difficult to crack at low temperatures.^31,32^ As a result, the conversion of fuel-N to volatile-N was significantly higher for biomass than for coal at lower temperatures. Under high-temperature pyrolysis conditions, HCN formation is positively correlated with volatile matter content; thus, SD featuring high volatile content exhibited a higher HCN conversion compared to LC. Notably, NH_3_ is primarily derived from the hydrogenation of N-Q.^24^ As the N-Q content in LC is higher than in SD, the coal demonstrated a higher conversion rate to NH_3_ at elevated temperatures.

**Fig. 3:**
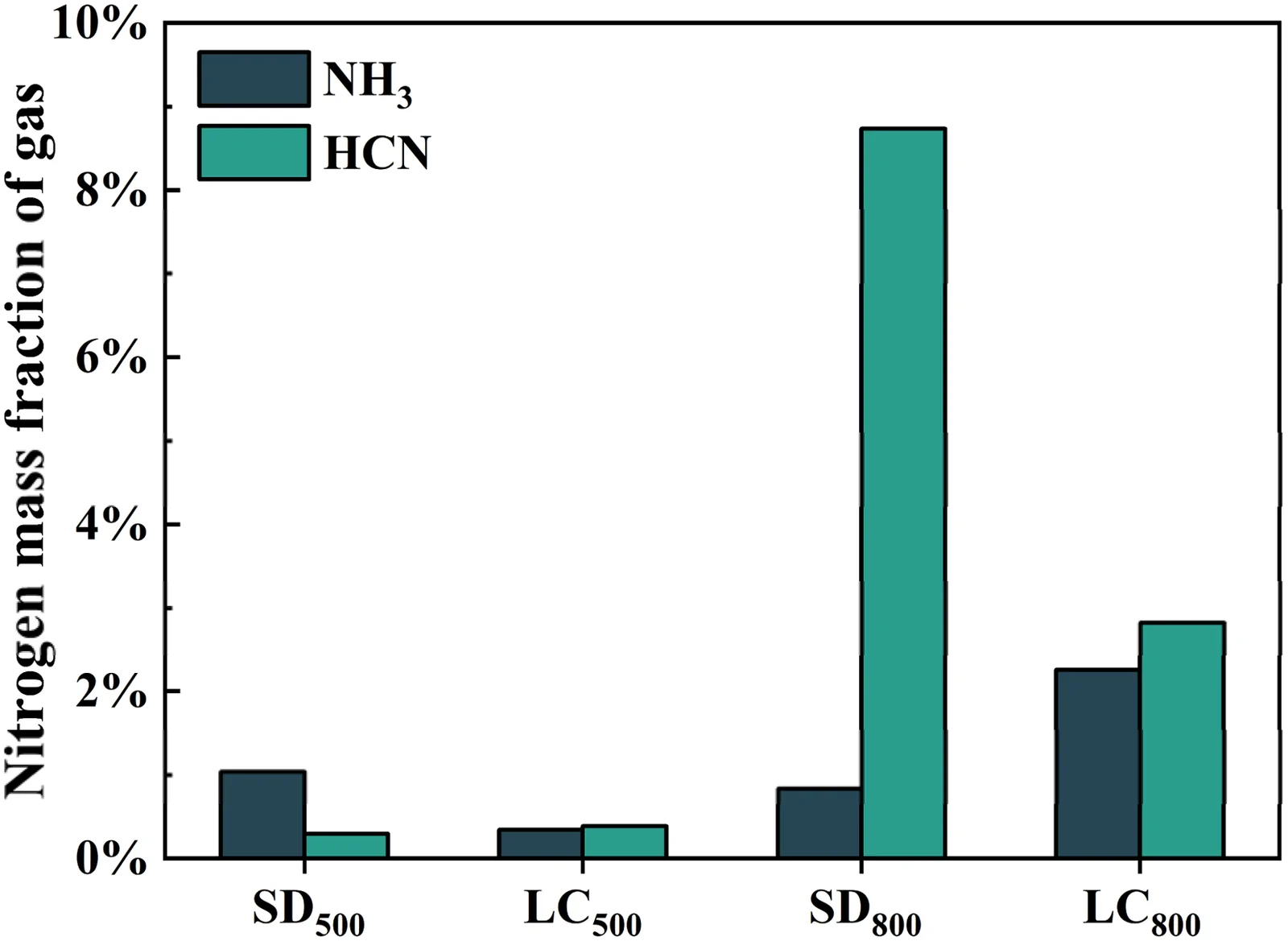
Volatile-N yield of SD and LC during fast pyrolysis at 500 °C and 800 °C.

#### 3.1.2 Evolution of Char-N structures

In this study, the assignment of binding energies to specific nitrogen forms and the subsequent peak deconvolution were conducted based on established literature.^10,33^ The primary nitrogenous component in biomass is identified as N-A, whereas N-5 is the dominant form in coal.^34^Fig. 4 presents the XPS (N 1s) spectra of the samples and their chars, and Fig. 5 illustrates the quantitative results of these nitrogen functional groups.

**Fig. 4:**
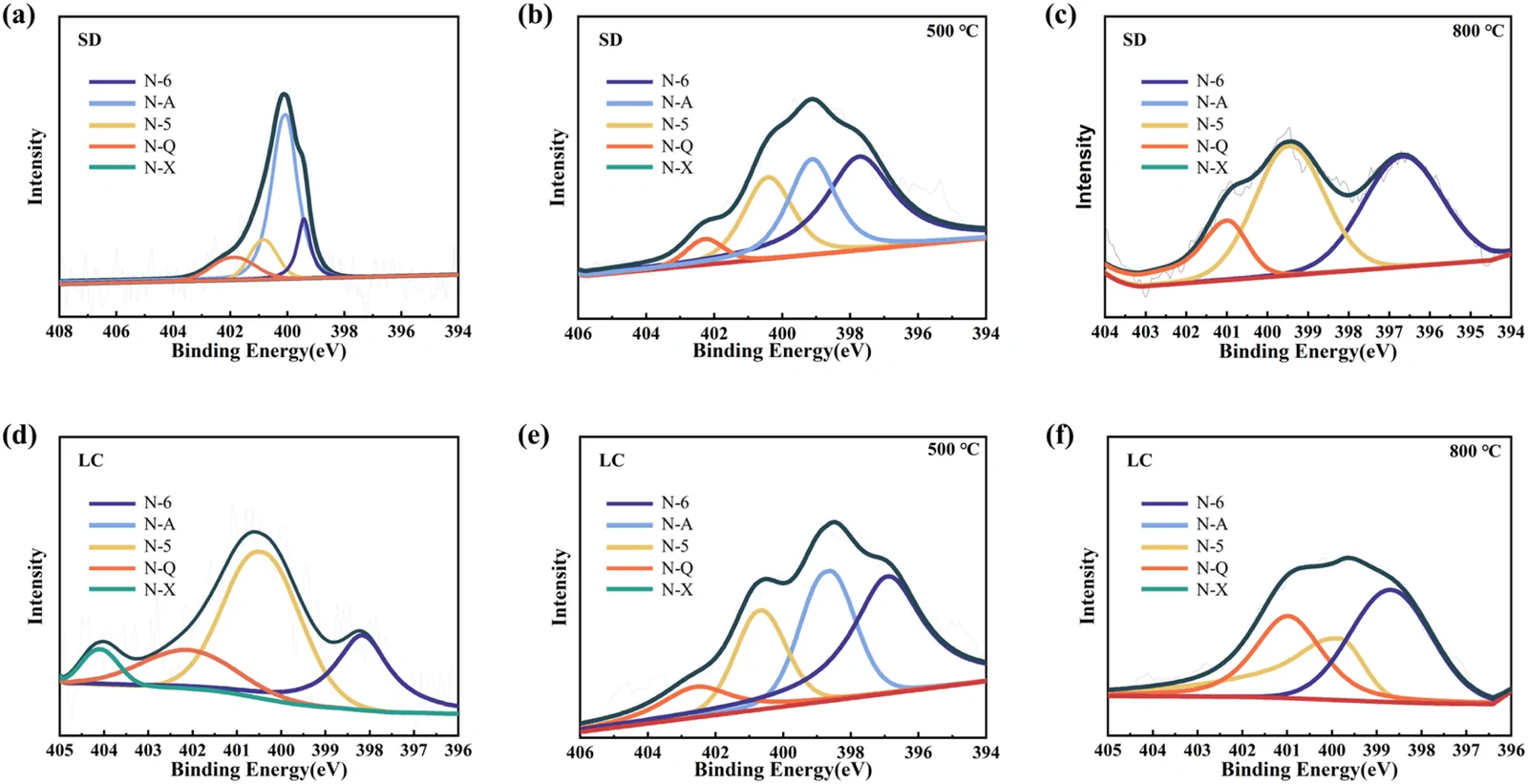
N 1s XPS spectra of raw samples and the pyrolyzed char. (a) SD, (b) SD_500_, (c) SD_800_, (d) LC, (e) LC_500_, and (f) LC_800_.

**Fig. 5:**
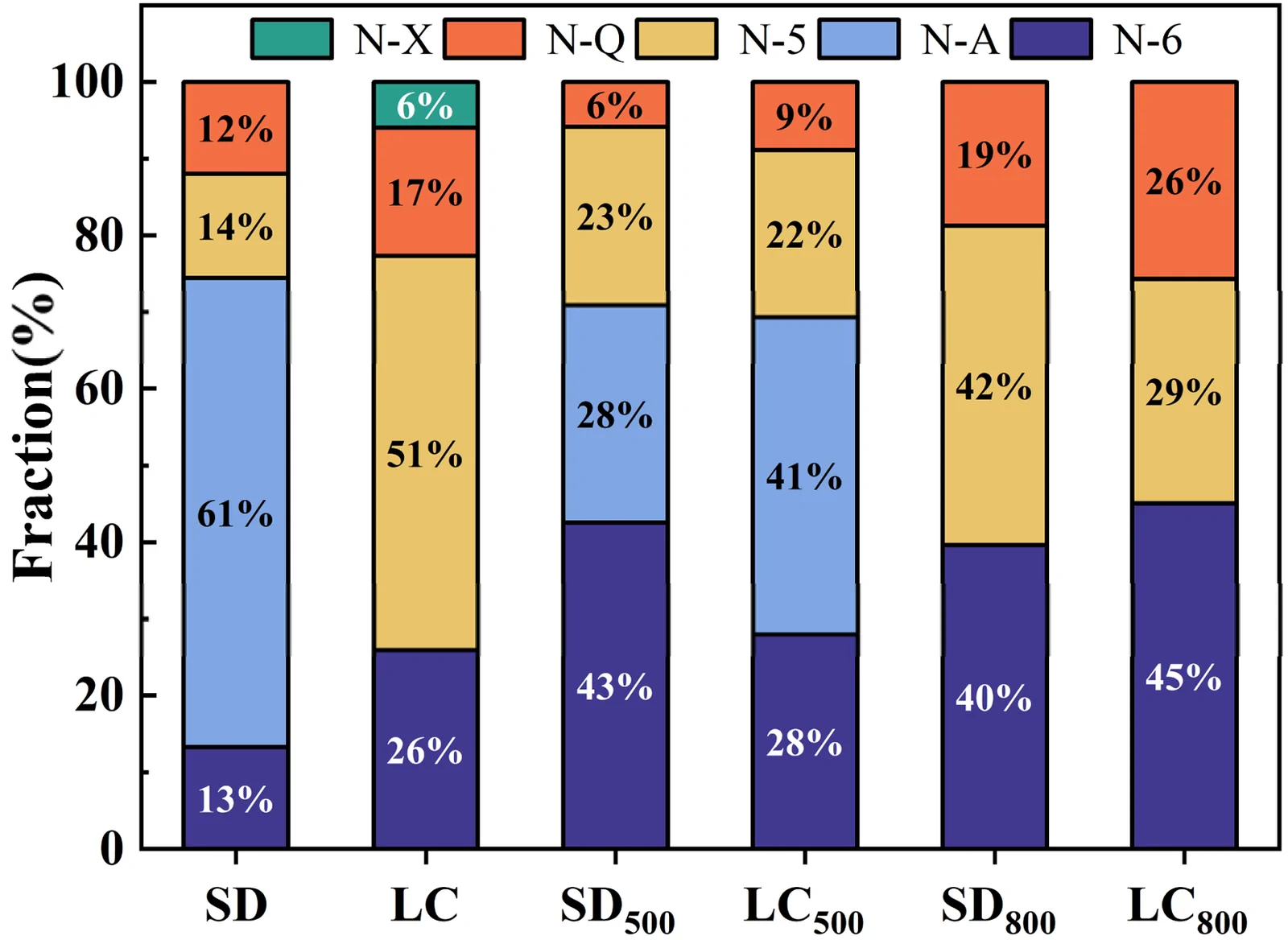
Relative content variations of nitrogen species in the raw samples and pyrolyzed chars.

As shown in Fig. 4a and d, the XPS spectra of SD exhibited a single peak corresponding to N-A, while LC displayed one main peak and two additional shoulder peaks, indicating that the predominant structure was N-5, accompanied by a small amount of N-X.^10^ During rapid pyrolysis at 500 °C, the N-A in SD char decreased significantly, with N-6 becoming the dominant char-N structure at 43% (Fig. 4b). This transformation occurs because thermally labile N-A converts into volatile-N, while the relatively stable fraction of N-A transforms into N-5 and N-6 *via* condensation reactions. In contrast, for the LC sample pyrolyzed at 500 °C, the relative content of N-5 decreased, whereas N-6 and N-Q increased slightly (Fig. 4e). Since the N-A in biomass decomposes at lower temperatures, whereas the heterocyclic nitrogen rings in coal are thermally stable, the variation in char-N distribution was substantial for SD but insignificant for LC under low-temperature conditions (500 °C).

During fast pyrolysis at 800 °C, the N-A in SD completely decomposed (Fig. 4c). The resulting char-N distribution was dominated by N-5 (42%) and N-6 (40%). Mechanistically, thermally labile N-A is released as volatile-N, while relatively stable N-A is converted into N-5 and N-6 *via* condensation reactions. At high temperatures, a portion of N-5 is released as volatiles (specifically HCN) *via* ring-opening reactions, while the remainder converts into N-Q and the more thermally stable N-6.^24,31^ As for the LC char pyrolyzed at 800 °C, as shown in Fig. 4f, char-N consisted of N-5, N-6, and N-Q, while N-X was completely volatilized.^35^ As shown in Fig. 5, the relative content of N-5 in LC decreased from 51% (raw samples) to 29%, whereas N-6 increased from 26% to 45%, and N-Q increased from 17% to 26%. This suggests a conversion of N-5 into N-6 and N-Q with increasing temperature. Note that the N-Q in raw coal is typically associated with pyridinic structures involving hydroxyl groups, whereas char-N in the form of N-Q represents nitrogen incorporated in the graphitic layers through heterocyclic six-membered rings, possessing higher stability.^36^

In summary, during low-temperature pyrolysis (500 °C), the labile N-A in SD decomposes to generate NH_3_, while the stable N-A converts into heterocyclic char-N *via* condensation, serving as a precursor for high-temperature HCN formation.^24,34^ Due to the stability of heterocyclic rings at low temperatures, SD releases a significant amount of NH_3_ but only trace HCN, whereas coal releases minimal volatile-N with little change in char-N composition. During high-temperature fast pyrolysis (800 °C), the labile N-A in SD continues to release NH_3_. Meanwhile, stable N-A converts into heterocyclic nitrogen, and a larger proportion converts to N-5 than during low-temperature pyrolysis. These heterocyclic structures further decompose to form HCN or gradually transform into stable N-Q. In LC, heterocyclic nitrogen first undergoes ring-opening driven by radicals to form HCN, followed by a gradual transformation into N-Q. The N-Q in coal also undergoes slow hydrogenation to form NH_3_. Consequently, compared to SD, pyrolysis of LC favors the solid-phase transformation of N-5 into N-6 and N-Q, resulting in lower HCN emissions.

### 3.2 Effect of Na on nitrogen evolution during individual pyrolysis of biomass/coal

Fig. 6a illustrates the distribution of volatile-N species (NH_3_/HCN) during the pyrolysis of SD and SD-Na at 500 °C and 800 °C. Under low-temperature pyrolysis conditions (500 °C), the addition of Na increased the yields of both apparent NH_3_ and HCN. In contrast, at high temperatures of 800 °C, the presence of Na resulted in a reduction of both NH_3_ and HCN yields. These results indicate that the addition of 2 wt% NaCl promotes the formation of NH_3_ and HCN at lower temperatures but inhibits their generation at higher temperatures. This finding aligns with the results reported by Zhao *et al.*,^35^ who demonstrated that Na addition reduced the total release of HCN/NH_3_ at high temperatures. As for the pyrolysis of LC and LC-Na (Fig. 6b), the conversion ratios of fuel-N to both NH_3_ and HCN were slightly lower for the LC-Na sample than for LC at 500 °C. While at a pyrolysis temperature of 800 °C, the yields of NH_3_ and HCN released from coal decreased with Na loading, a trend consistent with that observed for biomass. These results indicate that the presence of Na inhibits the evolution of NH_3_ and HCN during coal pyrolysis at both 500 °C and 800 °C.

**Fig. 6:**
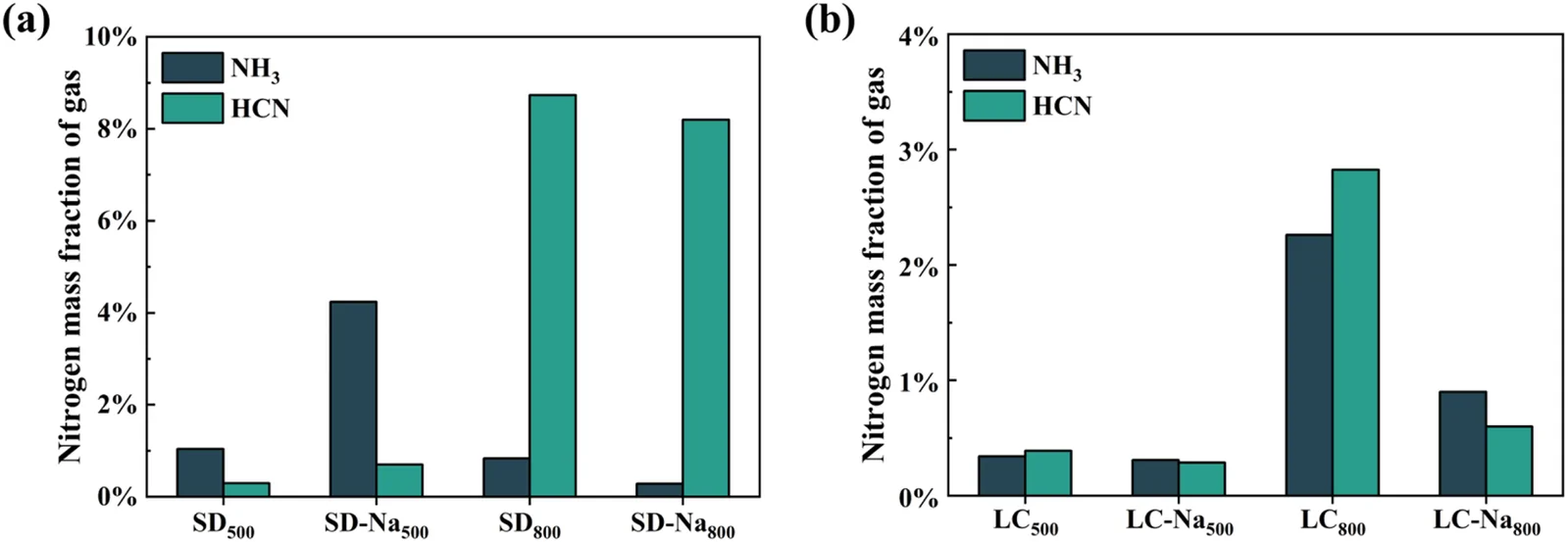
Volatile-N yield of (a) SD and SD-Na and (b) LC and LC-Na pyrolysis at 500 °C and 800 °C.

Fig. 7 presents the XPS (N 1s) spectra of the chars obtained from SD and SD-Na pyrolysis at 500 °C and 800 °C, while Fig. 8 details the specific distribution of these nitrogen functional groups. As depicted in Fig. 8, distinct variations in char-N distribution were observed depending on the pyrolysis temperature. Under low-temperature conditions (500 °C), the char derived from SD-Na exhibited a higher relative content of N-A (40%) and a lower relative content of N-6 (16%) compared to the char from SD. At a high temperature of 800 °C, the SD-Na char showed a decrease in the relative content of N-5 and an increase in N-Q compared to the SD char. According to previous studies,^35^ the addition of alkali metals such as Na significantly influences the interconversion of nitrogen functional groups within the char matrix. At low pyrolysis temperatures, Na facilitates thermal decomposition of labile N-A (such as amides) to release amino groups, which subsequently form NH_3_, while simultaneously promoting the condensation of stable N-A into heterocyclic nitrogen.^34,37,38^ At relatively high temperatures, the presence of Na inhibits ring-opening and hydrogenation of less stable heterocyclic nitrogen structures in the char, thereby reducing volatile-N formation and catalyzing the transformation of the less stable N-5 into the more stable N-Q.

**Fig. 7:**
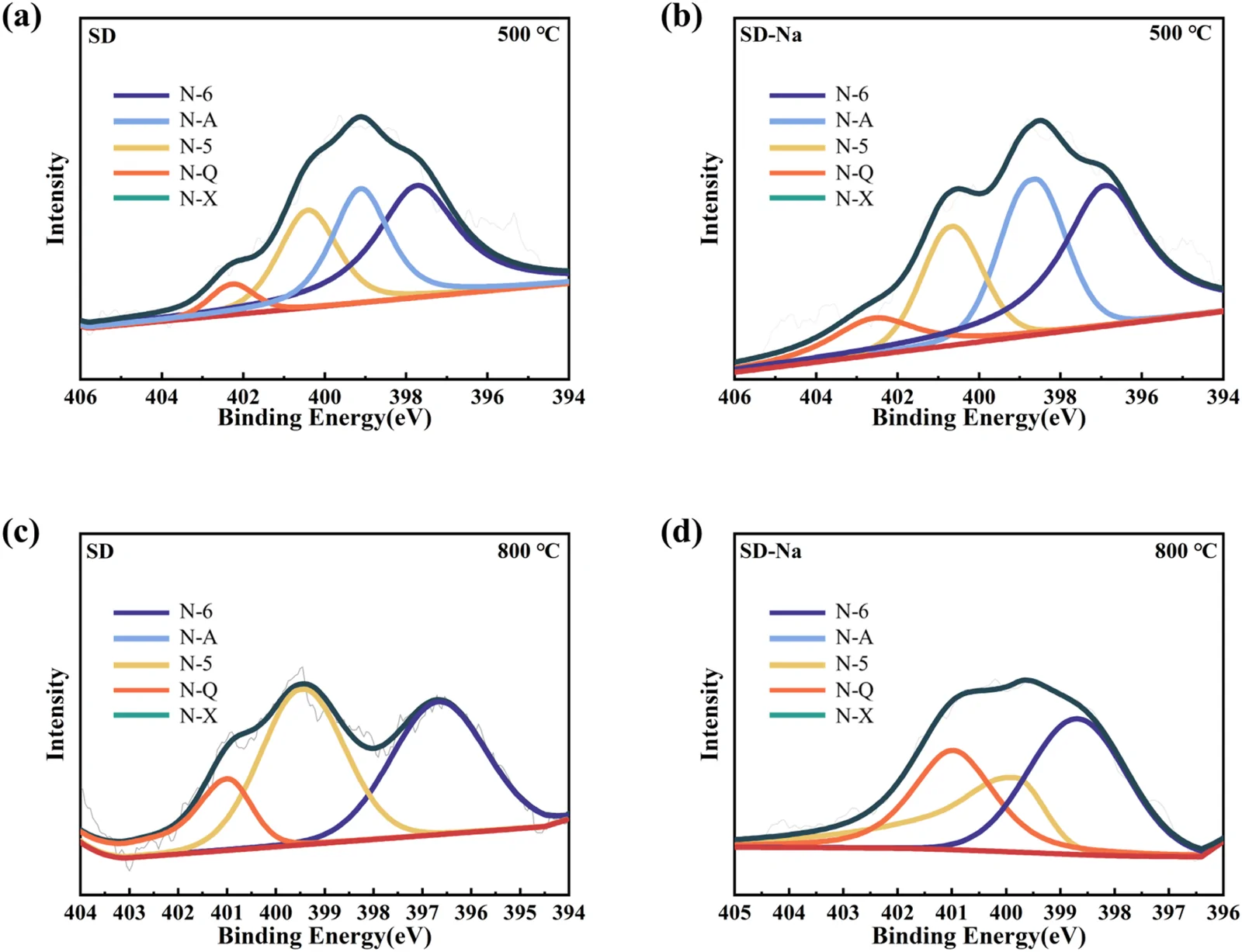
N 1s XPS spectra of SD and SD-Na pyrolysis at 500 °C and 800 °C. (a) SD_500_, (b) SD-Na_500_, (c) SD_800_, and (d) SD-Na_800_.

**Fig. 8:**
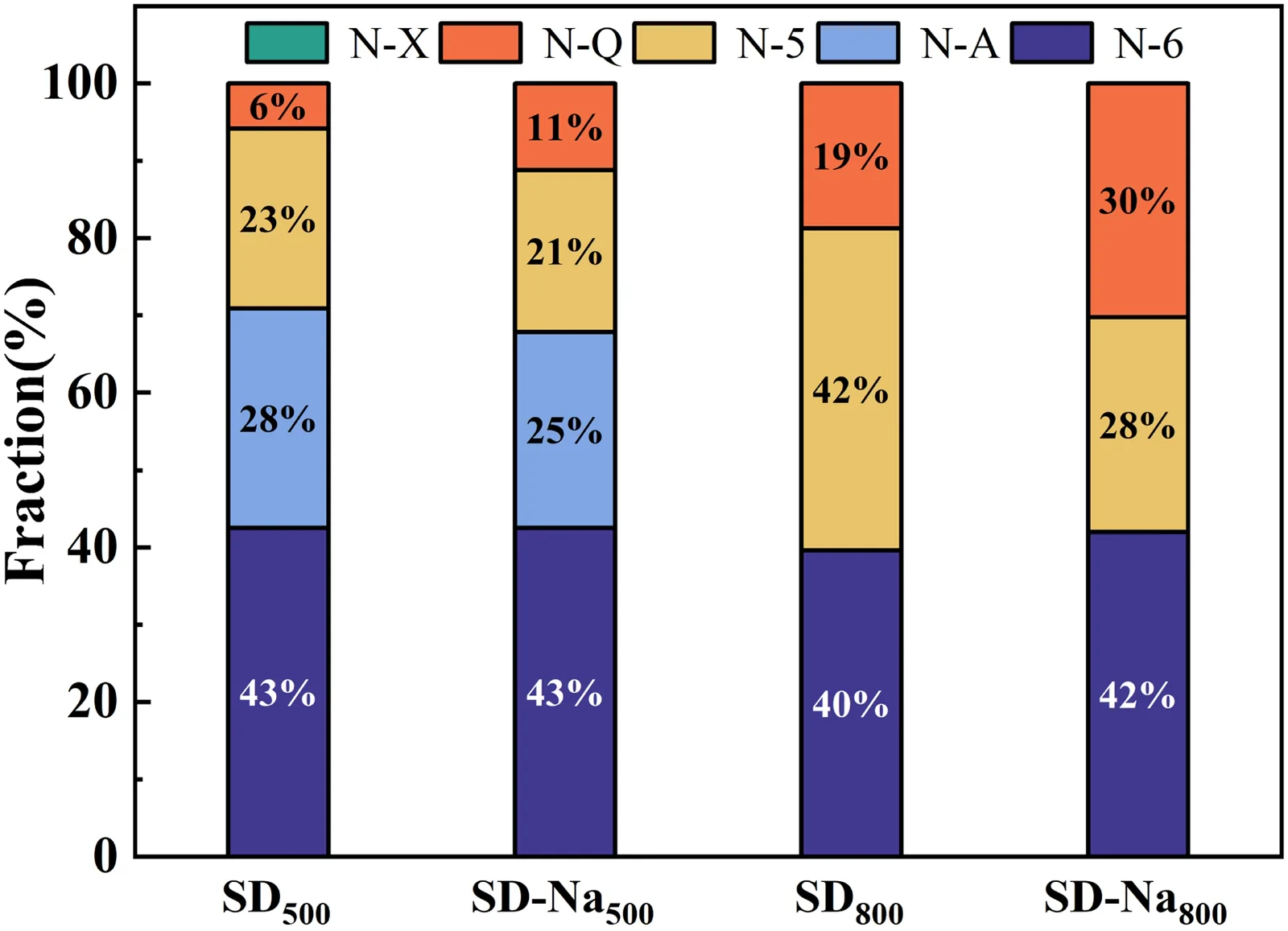
Relative content variations of nitrogen functional groups in SD and SD-Na pyrolysis at 500 °C and 800 °C.

### 3.3 Effect of Na on nitrogen evolution during rapid co-pyrolysis of biomass/coal

Fig. 9 illustrates the distribution of NH_3_ and HCN released during the pyrolysis of blended samples at 500 °C and 800 °C. These blends consist of acid-washed LC mixed with either acid-washed SD or 2 wt% NaCl-impregnated acid-washed SD-Na at biomass blending ratios of 5% and 15%. As shown in Fig. 9a, under low-temperature pyrolysis conditions (500 °C), the addition of Na decreased the release of both NH_3_ and HCN at both the 5% and 15% blending ratios. Similarly, under high-temperature conditions (800 °C, Fig. 9b), the yields of NH_3_ and HCN from the 5SD/LC and 15SD/LC blends also decreased upon the addition of Na. These results indicate that the presence of Na consistently inhibits the release of volatile-N species during co-pyrolysis at both low and high temperatures.

**Fig. 9:**
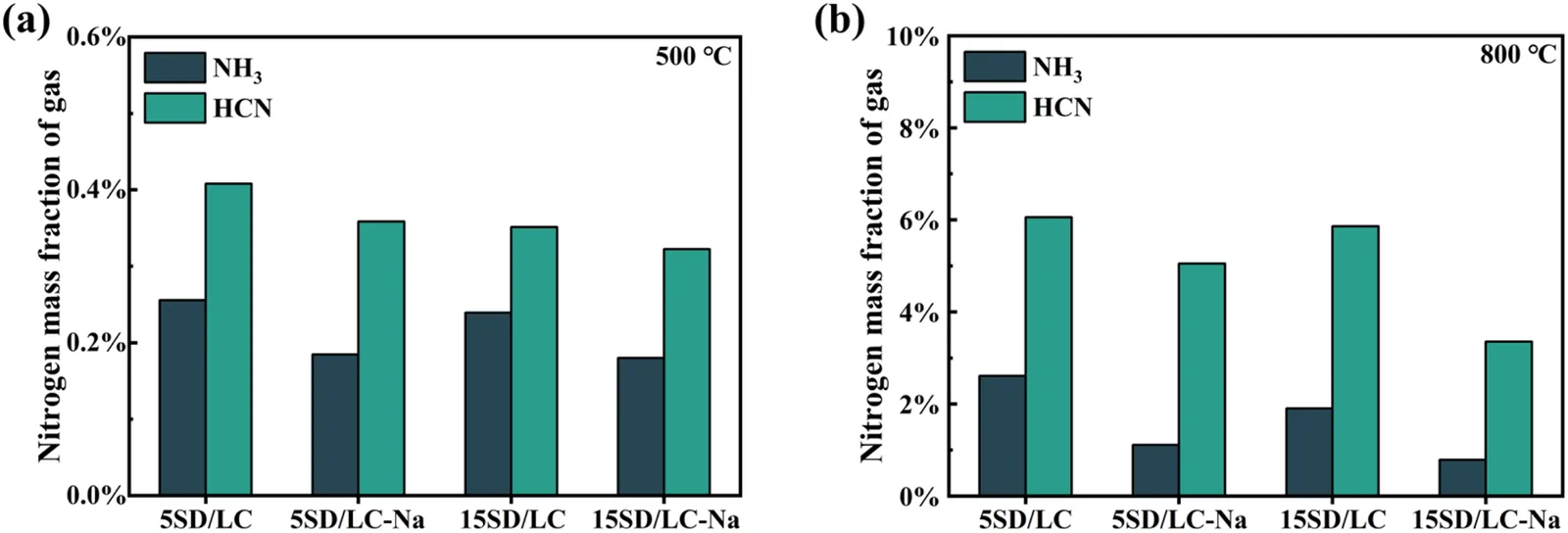
Volatile-N yield of blended samples. Pyrolysis at (a) 500 °C and (b) 800 °C.

Fig. 10 and 11 present the XPS (N 1s) spectra of the chars obtained from co-pyrolysis of the aforementioned 5% and 15% blends at 500 °C and 800 °C. Fig. 12 details the corresponding distribution of nitrogen functional groups within these chars. Under low-temperature co-pyrolysis (Fig. 10), the char-N in the blends is predominantly composed of N-5 and N-6. For the 5% blend, the addition of Na increased the relative content of N-5 (from 32% to 41%) and N-6 (from 16% to 28%), while decreasing the relative content of N-A (from 8% to 6%) compared to the Na-free blend. For the 15% blend, Na addition similarly elevated the N-5 content (from 34% to 43%), decreased the N-A content (from 13% to 10%), and slightly increased the N-6 content. While under high-temperature co-pyrolysis (800 °C, Fig. 11), the char-N in all blends is dominated by N-5. For the 5% blend, Na addition significantly increased the relative content of N-5 (from 40% to 56%) and slightly increased quaternary nitrogen (N-Q) (from 23% to 26%), while substantially reducing N-6 (from 37% to 18%). For the 15% blend, the addition of Na slightly increased N-5 (from 39% to 45%) and N-Q (from 27% to 33%), whereas N-6 decreased (from 34% to 21%). The opposite response of N-6 at the two temperatures reflects a change in the controlling step. At 500 °C, the Na-promoted condensation of N-A into heterocyclic nitrogen still proceeds and N-6 accumulates, whereas at 800 °C, this source is exhausted and N-6 is consumed by ring-opening and conversion into N-Q, assisted by the catalytic decomposition of the large aromatic structures hosting it.

**Fig. 10:**
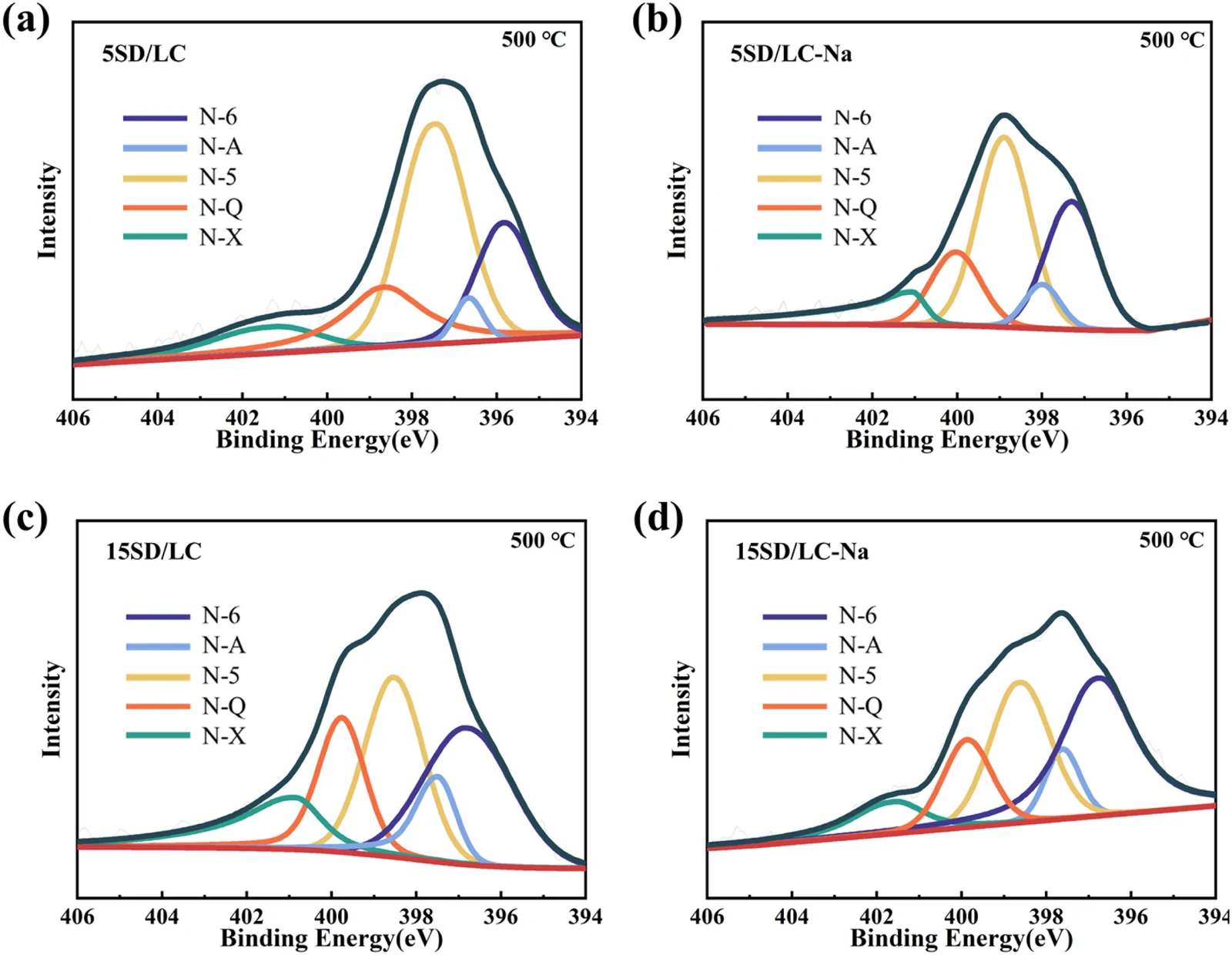
N 1s XPS spectra of blended sample char at 500 °C (a) 5SD/LC_500_, (b) 5SD/LC-Na_500_, (c) 15SD/LC_500_, and (d) 15SD/LC-Na_500_.

**Fig. 11:**
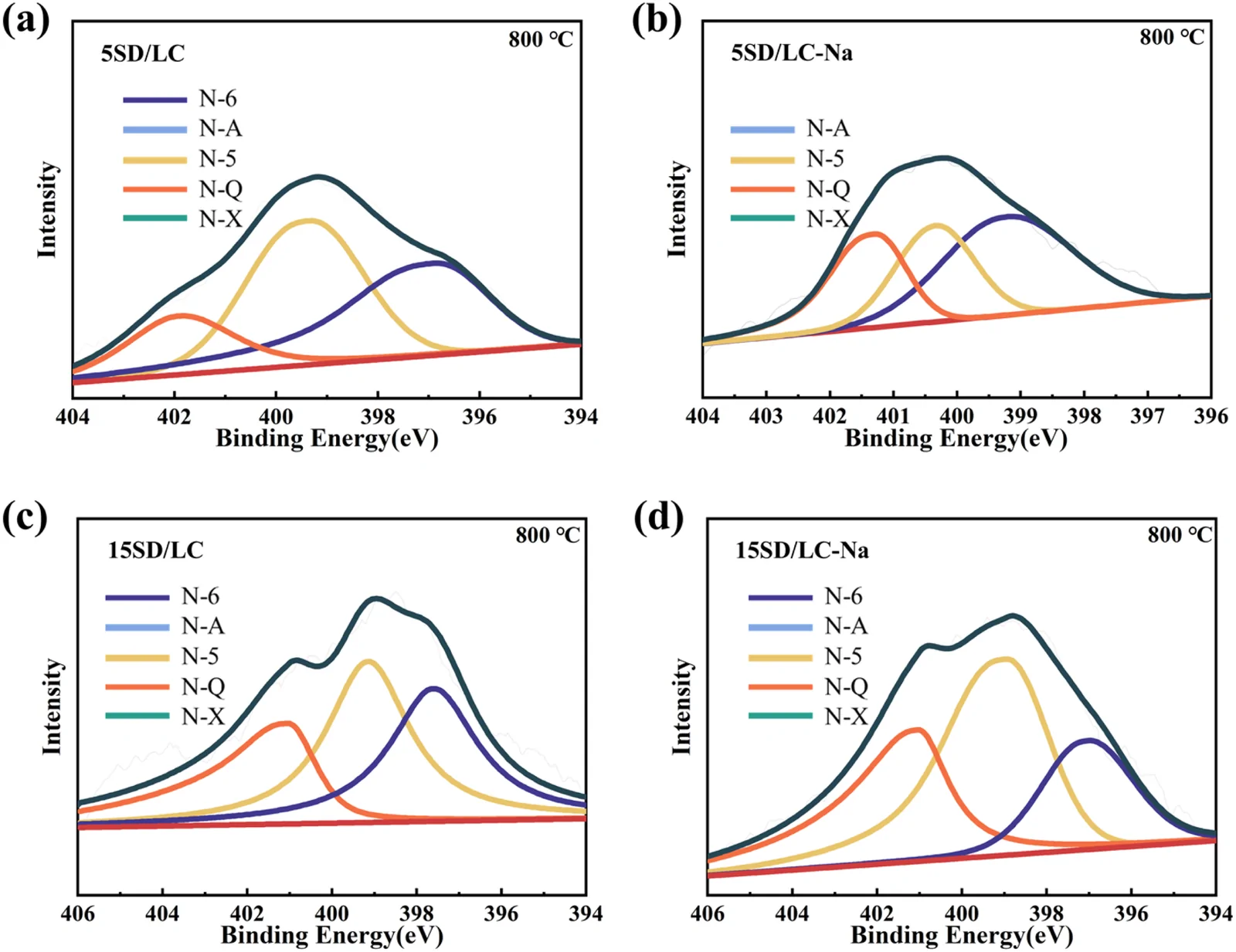
N 1s XPS spectra of blended sample char at 800 °C (a) 5SD/LC_800_, (b) 5SD/LC-Na_800_, (c) 15SD/LC_800_, and (d) 15SD/LC-Na_800_.

**Fig. 12:**
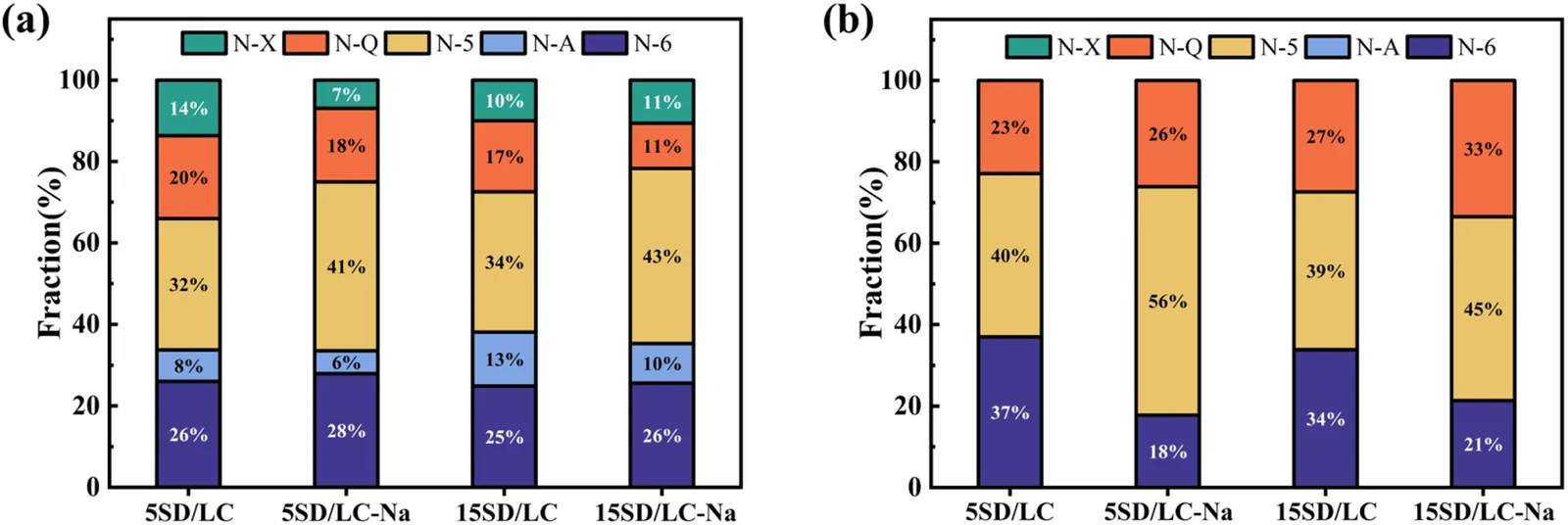
Distribution of nitrogen species in blended sample char at (a) 500 °C and (b) 800 °C.

Fig. 13 illustrates the Raman spectral deconvolution results for chars obtained from co-pyrolysis of the blended samples at 500 °C and 800 °C. The data specifically highlights the ratio of small to large aromatic rings (*A*_V_R_+V_L_+G_R__/*A*_D_) and the relative proportion of small aromatic rings within the total carbon structure (*A*_V_R_+V_L_+G_R__/*A*_all_). Upon loading 2 wt% NaCl, chars produced from the co-pyrolysis of SD and LC (at both 5% and 15% blending ratios) exhibited an increased ratio of small to large aromatic rings, alongside an overall increase in the proportion of small aromatic rings. Mechanistically, during the co-pyrolysis process, Na-containing volatiles released from the biomass migrate through the gas phase and deposit on the surface of coal particles. Such surface deposition has been widely reported for the co-pyrolysis of coal with lignocellulosic biomass.^39,40^ The capture of released Na species is favored by the abundant oxygen-containing groups of coal, which provide ion-exchange sites for the formation of C–O–Na structures on the char surface, governing the subsequent catalytic behavior. Notably, such deposition is further favored in the present fixed-bed reactor, where volatiles released from the biomass must percolate through the packed blend and contact the coal particles before reaching the sweep gas.^41^ On the one hand, the presence of Na inhibits both the release of volatile-N and the polycondensation of small aromatic rings,^42^ so that the char-N was redistributed towards N-5, as indicated by XPS analysis. On the other hand, Na facilitates the formation of stable complexes between small aromatic ring structures (3–5 rings) and PAHs,^43^ which subsequently hinders the transformation pathway from N-5 to N-6. Concurrently, the catalytic effect of Na accelerates the decomposition of large aromatic ring structures in the sample, ultimately leading to a decrease in the relative content of N-6.

**Fig. 13:**
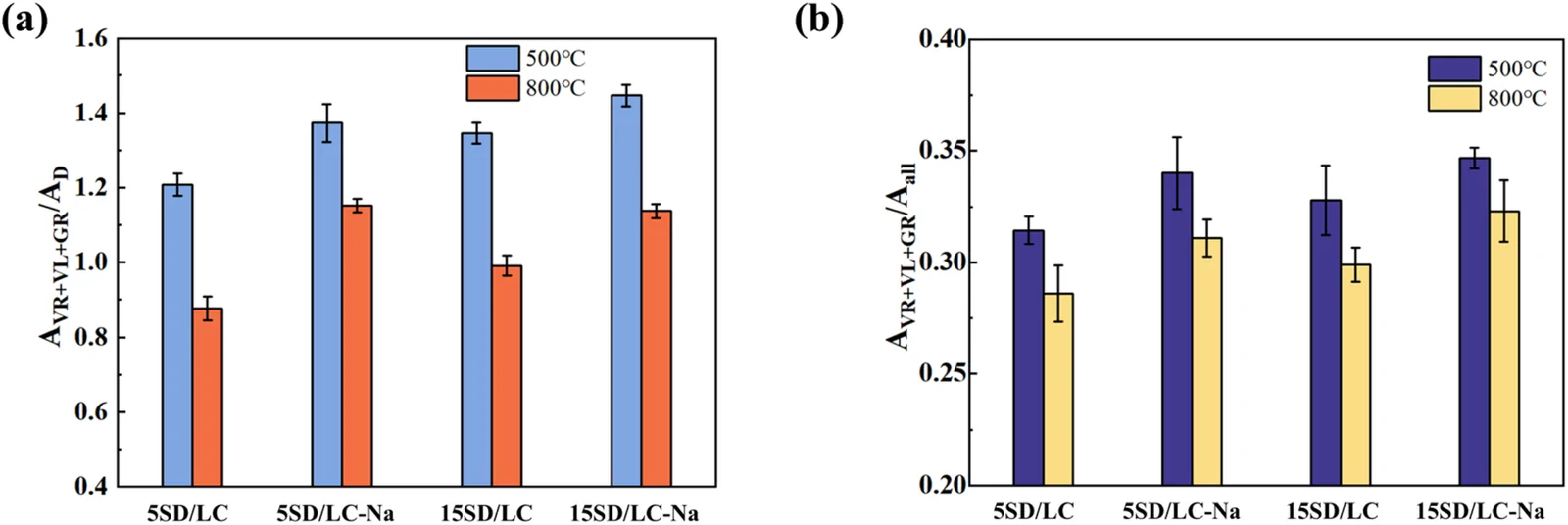
Raman-derived (a) *A*_V_R_+V_L_+G_R__/*A*_D_ and (b) *A*_V_R_+V_L_+G_R__/*A*_all_ of the chars from blended samples pyrolyzed at 500 °C and 800 °C.

This deposition can also be inferred from the char structure and nitrogen speciation. Since Na was introduced only into the biomass fraction, which accounted for 5% and 15% of the blends, the coal fraction dominates the mass of the resulting chars. Nevertheless, the Na-loaded blended chars exhibited increased *A*_V_R_+V_L_+G_R__/*A*_D_ and *A*_V_R_+V_L_+G_R__/*A*_all_ ratios (Fig. 13), a stabilization of char-N as N-5 and N-Q at the expense of N-6 (Fig. 12), and a suppressed release of NH_3_ and HCN at both temperatures (Fig. 9). These changes cannot be attributed to the biomass fraction alone, and therefore imply that Na became associated with the coal-derived char. This requires that Na introduced into the biomass was released from the biomass particles and subsequently retained by the coal-derived char, indicating that the volatilization and redeposition of Na occurred under the present conditions. Beyond this qualitative inference, the coupling between the modified carbon skeleton and nitrogen release was further quantified by relating the Raman-derived proportion of small aromatic rings to the total volatile-N yield for the four blended samples at 500 and 800 °C (Fig. 14). At both temperatures, the volatile-N yield decreased as *A*_V_R_+V_L_+G_R__/*A*_all_/*A*_D_ (Fig. 14a) increased, with correlation coefficients of 0.975 at 500 °C and 0.887 at 800 °C; the same relationship was obtained using *A*_(V_R_+V_L_+G_R_)_/*A*_all_ (0.996 and 0.968), as shown in Fig. 14b. This relationship holds within each temperature, which suggests that polycondensation of the carbon skeleton and suppressed release of volatile-N are intrinsically coupled, and confirms that Na regulates nitrogen conversion through its modification of the aromatic structure.

**Fig. 14:**
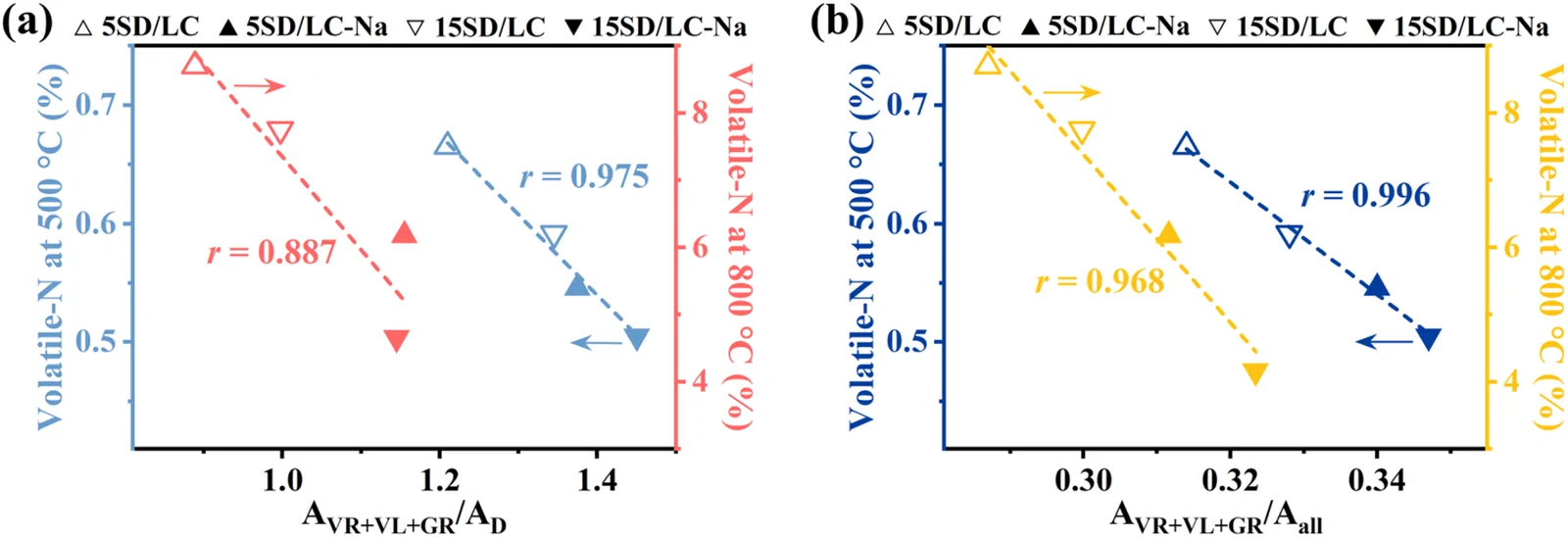
Relationship between the Raman-derived proportion of small aromatic rings and the total volatile-N yield of the blended chars at 500 °C and 800 °C. (a) *A*_V_R_+V_L_+G_R__/*A*_D_, and (b) *A*_V_R_+V_L_+G_R__/*A*_all_.

Based on the above results, the effect of Na on nitrogen evolution during the fast co-pyrolysis of SD and LC can be rationalized by coupling volatile-N release, char-N transformation, and carbon skeleton evolution. During individual pyrolysis, the labile N-A in SD is readily decomposed at low temperatures to form NH_3_, whereas the heterocyclic nitrogen structures in LC, mainly including N-5 and N-6, remain relatively stable. At elevated temperatures, these heterocyclic nitrogen species may undergo ring-opening to generate HCN or be gradually transformed into more stable N-Q structures. After introducing Na into the blended samples, Na-containing volatile species released from SD can migrate and deposit onto the surface of coal particles during co-pyrolysis,^39^ thereby modifying the local char matrix and nitrogen conversion pathways.

For blended samples, Na consistently suppresses the release of NH_3_ and HCN at both 500 °C and 800 °C, indicating that Na inhibits the conversion of nitrogen-containing intermediates into volatile-N species during co-pyrolysis. This inhibition can be attributed to two coupled effects. First, Raman analysis shows that Na increases the ratio of small to large aromatic rings and the relative proportion of small aromatic structures in the co-pyrolysis chars, suggesting that Na inhibits excessive polycondensation of the carbon skeleton. Second, within such a modified aromatic carbon matrix, the ring-opening of heterocyclic nitrogen to HCN is suppressed, while the hydrogenation of N-Q to NH_3_ is restrained through the limited availability of donatable hydrogen, so that char-N is stabilized in the form of heterocyclic nitrogen species, particularly N-5 and N-Q. As shown in Fig. 14, these two effects are coupled rather than independent; hindered polycondensation of the carbon skeleton and suppressed release of volatile-N vary together across all blended conditions at each temperature. Therefore, as illustrated in Fig. 15, Na reduces the formation of gaseous NO_*x*_ precursors by suppressing volatile-N release and redirecting char-N towards more stable heterocyclic forms during the fast co-pyrolysis of coal and biomass.

**Fig. 15:**
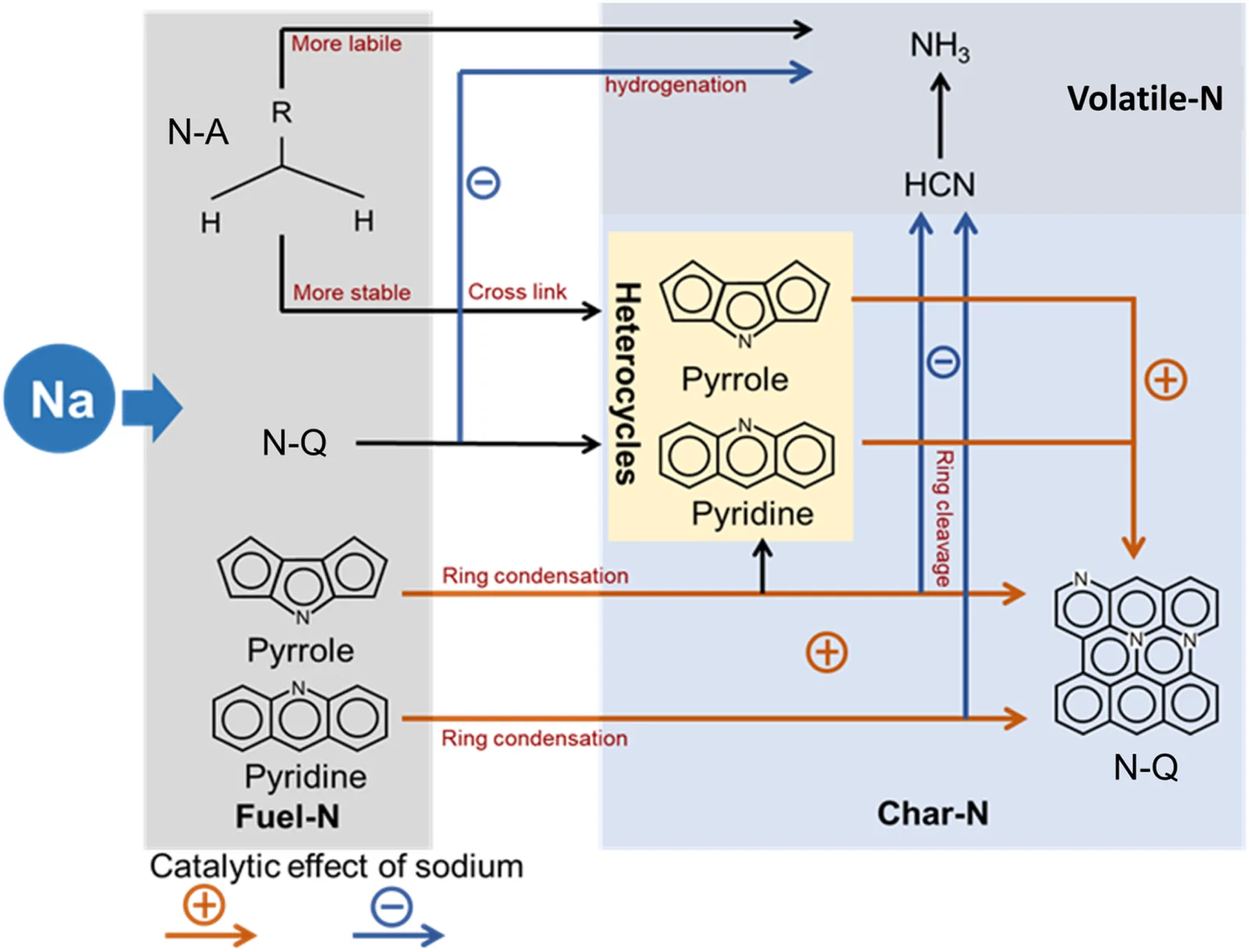
Proposed mechanism for the effect of Na on volatile-N release and char-N stabilization during the fast co-pyrolysis of coal and biomass.

## 4. Conclusions

This study investigated the migration and transformation of fuel-N during rapid pyrolysis and co-pyrolysis of sawdust (SD) and lignite coal (LC) at 500 °C and 800 °C, using acid-washed and Na-loaded samples to clarify the regulatory role of Na. The two fuels followed distinct nitrogen evolution pathways: in SD, the labile N-A underwent thermal decomposition to release NH_3_, while the stable N-A converted into heterocyclic char-N *via* condensation; whereas in LC, the heterocyclic nitrogen required ring-opening to form HCN, so that the pyrolysis of LC favored the solid-phase transformation of N-5 into N-6 and N-Q and resulted in lower volatile-N release. For individual pyrolysis of SD, the addition of Na promoted the formation of NH_3_ and HCN at 500 °C but inhibited their generation at 800 °C, while for the pyrolysis of LC and all blended samples, Na inhibited the evolution of NH_3_ and HCN at both 500 °C and 800 °C, indicating that Na suppressed the conversion of fuel-N to the gaseous NO_*x*_ precursors. This inhibition originated from two coupled effects: Na suppressed the ring-opening of heterocyclic nitrogen into HCN and the hydrogenation of N-Q into NH_3_, and Raman analysis showed that Na inhibited the polycondensation of small aromatic rings, with the volatile-N yield of the blended chars decreasing monotonically as the proportion of small aromatic rings increased at both temperatures, so that the char-N was redistributed towards N-5 and N-Q. These findings indicate that alkali metals in co-fired systems act not only as a source of slagging and fouling, but also as an active regulator of gaseous NO_*x*_ precursor formation. In particular, demineralization pretreatments commonly adopted to mitigate ash-related problems may therefore inadvertently enhance the release of NH_3_ and HCN so that alkali metal management in biomass-coal co-firing involves a trade-off between ash behavior and the suppression of primary NO_*x*_ precursor formation.

## Author contributions

The manuscript was written through contributions from all the authors.

## Conflicts of interest

The authors declare no conflicts of interest.

## Data Availability

The original contributions presented in this study are included in the article, and further inquiries can be directed to the corresponding author.
